# An Evaluation of the Retinal Nerve Fibre Layer and Ganglion Cell Layer Thickness in Low Myopes

**DOI:** 10.1007/s44402-026-00021-7

**Published:** 2026-03-13

**Authors:** AJ Munsamy, Lungile Mafynn Buthelezi, Lindokuhle Zama Biyela, Bibi Haajirah Kaja, Sthembile Felicia Magubane, Kairav Mahabeer, Lelethu Mahamba, Sfundo Mzikayifani Nkosi, Nomaswazi Nomaqhawe Vumase, Nonduduzo Bonelile Zondo, Sisanda Vuyiswa Zulu

**Affiliations:** Discipline of Optometry, School of Health Science, University of KwaZulu-Natal, Durban, South Africa

**Keywords:** African, Ganglion cell layer, Myopia, OCT, Retinal nerve fibre layer, Retinal thickness

## Abstract

**Introduction:**

To evaluate the integrity of the peripapillary (pp) and macular (m) retinal nerve fibre layer (RNFL) and the macular ganglion cell layer (GCL) in young adults with low myopia.

**Methodology:**

The study was observational and cross-sectional in design. A total of 122 participants (61 emmetropes and 61 low myopes) were recruited, with a mean age 20.5 ± 1.86 years. Low myopia was defined as a spherical equivalent refraction (SER) between −0.75 and −3.00 D; controls had SER between +0.50 and −0.50 D. Refractive error was measured using the Essilor AKR550 Auto Kerato-Refractor. Axial length (AL) was assessed with the NIDEK AL-Scan Optical Biometer. Retinal layers were imaged using the Heidelberg Spectralis OCT, capturing macular RNFL (mRNFL), peripapillary RNFL (ppRNFL), Bruch’s membrane opening-RNFL (BMO-RNFL) and macular GCL (mGCL) thickness. Independent *t* tests/Mann–Whitney *U*tests compared thicknesses; multivariate linear regression evaluated associations between AL, SER, RNFL and GCL measures. The Holm–Bonferroni correction was applied for all *p* values.

**Results:**

Mean SER was −0.25 ± 0.25 D (emmetropes) and −1.25 ± 0.57 D (low myopes); mean AL in the same groups was 23.41 ± 0.77 and 23.76 ± 0.82 mm, respectively. Low myopes showed significant thinning in the inferior (*p* = 0.03); Cohen’s *d* effect size = −0.23 and temporal (*p* = 0.01); Cohen’s *d* effect size = −0.61 regions of the outer mGCL. AL showed positive associations mostly with mRNFL and mGCL, although they were weaker in low myopes. Similarly, weaker positive correlations were also found in low myopes between AL and global ppRNFL (*p* = 0.02) when compared with near-emmetropes.

**Conclusion:**

Significant differences in the inferior and temporal GCL layers in low myopes may suggest early structural changes. These findings highlight that myopia is not solely a refractive issue and suggest early intervention strategies may not be limited to high myopia cases.

Key Points

- Even in young adults with low myopia, measurable thinning was found in key retinal layers—including the ganglion cell layer, indicating that structural changes may begin earlier than previously thought.
- These findings challenge the common belief that low myopia is innocuous, revealing that structural damage to the eye can occur even at mild levels of refractive error.
- Myopia control efforts may consider including young adults with low myopia, as early intervention could be critical to preserving long-term eye health.

## Introduction

Myopia is becoming a global pandemic with a prevalence of up to 80% in Asian countries [1]. The most cited cause of myopia progression is axial elongation of the globe, leading to stretching and thinning of the retina, with subsequent impairment to retinal function [2]. The International Myopia Institute (IMI) defined low myopia as a condition in which the spherical equivalent refractive error (RE) is ≤ −0.50 D and > −6.00 D when ocular accommodation is relaxed [3]. However, other studies have classified myopia as low when the magnitude is less than 3 D [4–7]. Moderate myopia is between 3 and 6 D, whilst high myopia is >6 D [4]. Low and high myopia are further classified by an axial length (AL) < 24 mm and >30 mm, respectively [4].

Myopia progression has been associated with several factors that include genetics and environmental factors, mode of correction and time spent on near tasks and outdoors [8]. These factors support the theories of myopia progression, which include the mechanical tension theory [9], hyperopic defocus [10] and increased accommodative lag [11]. Understanding the mechanisms of myopia progression assists in identifying the ideal myopia control treatment plan. However, to contextualise these mechanisms, it is important to examine population-specific prevalence patterns and risk factors, particularly in African cohorts where epidemiological trends differ from global norms.

Research on myopia prevalence in African school children reveals consistently low rates compared with global trends. A comprehensive meta-analysis of 24 studies covering 36,395 African children found an overall myopia prevalence of 4.6% [12]. This finding aligns with other reviews showing prevalence ranging from 2.7 to 16.05% across different African regions [13]. In South African children specifically, myopia prevalence was 2.9% when quantified using retinoscopy and 4.0% using autorefraction, with rates increasing to 9.6% at 15 years of age [14]. Key risk factors include older age (12–18 years), female gender, family history, prolonged near work and higher parental education levels [12–14]. Importantly, myopic Africans face an increased risk of developing primary open-angle glaucoma [15].

The retinal nerve fibre layer (RNFL) and ganglion cell layer (GCL) have a useful utility in early detection of conditions such as glaucoma progression using the Optical Coherent Tomographer (OCT). The widespread use of the OCT enables the evaluation of RNFL and GCL thicknesses, which provides valuable information regarding the role of the inner retina in myopia. Myopia is associated with a gradual thinning of the RNFL and GCL [16]. However, investigations have predominantly focused on moderate to high myopes, resulting in a paucity of empirical support regarding the structural integrity of these retinal layers in low myopia. In light of prevailing epidemiological trends, a foreseeable surge in the worldwide incidence of myopia and its associated impact on retinal health is anticipated. Consequently, there is a compelling need for early intervention to mitigate myopia progression and to assess the structural integrity of the peripapillary (pp) region and macula (m). Therefore, this study sought to evaluate the thickness of the peripapillary (ppRNFL) and macula RNFL (mRNFL) thickness, as well as the macula ganglion cell layer (GCL) in low myopes using the Heidelberg Spectralis OCT.

## Materials and Methods

The study was a cross-sectional and observational design conducted at the University of KwaZulu-Natal eye clinic in Durban, South Africa. The study population comprised university students between the ages of 18–30 years, non-selective of ethnicity. Participants were recruited using the non-probability purposive sampling technique comprising an experimental group (low myopes) and a comparative group (near-emmetropes).

The anticipated sample size was 128, of which 64 participants were in the experimental group and 64 in the control group. The sample size determination formula adopted for this study was the two-sample *t* test: $n=\frac{({\sigma }_{1}^{2}\,+\,{\sigma }_{2}^{2}){(Z(\alpha /2)+Z(\beta ))}^{2}}{{\Delta }^{2}}$, where *n* is the sample size; *Z* is the standard normal distribution, equal to 1.96 for a 95% confidence level and α is the significance level and is equal to 0.05/5%. *σ* is the standard deviation. A 23.75 µm standard deviation of the ppRNFL measurements of low myopia, 8.5 µm standard deviation of the ppRNFL measurements of the control group and 9.82 µm expected mean of ppRNFL measurements difference between low myopia and control group were assumed for this study.

### Screening Tests

All participants underwent a series of preliminary tests to assess their eligibility for participation in this study. The screening procedure included a detailed case history, autorefraction/keratometry to determine RE and corneal curvature with the Essilor AKR550 Auto Kerato-Refractometer (https://essilorinstruments.co.za), slit lamp biomicroscopy and fundus photography to rule out any anterior and posterior segment pathology, respectively. The macula visual field was tested using the Amsler grid.

Monocular Estimate Method (MEM) retinoscopy was performed at 40 cm to measure the accommodative response of each participant, and best-corrected visual acuity (BCVA) was obtained from subjective refraction. Subsequently, intraocular pressure values were determined with the NIDEK NT530P Tonometer (https://www.nidek-intl.com).

### Selection Criteria

The control group comprised participants with autorefraction spherical equivalent (SE) between +0.50 and −0.50 D, whilst the experimental group consisted of SE autorefraction between −0.75 and −3.00 D, to fulfil the operational definition of low myopia [3]. Participants with astigmatism >0.50 D were excluded. To guard against pseudomyopia, participants with an accommodative response less than +0.25 D and greater than +0.50 D were excluded from this study [17]. Only participants with BCVA of 6/6 or better; no known or observed pathology, no macula visual field changes and an intraocular pressure (IOP) < 21 mmHg were included. The groups were sex matched.

### Data Collection

#### RE and biometry measurements

Each participant’s RE was measured objectively. Three readings for each eye were obtained, and the average taken as the final RE. Refraction data were transformed to spherical equivalent refraction (SER), i.e., sphere plus half cylinder power, and only the eye with the least myopic RE was used for OCT and AL measurements. The AL was measured using the Ocular Master (Zeiss; https://www.oculus.de), and an average of six automatic measurements was obtained. The AL-Scan incorporated auto-tracking and auto-shot accuracy of the AL measurements. The eye with the least myopic refraction was considered for data analysis to avoid inter-eye correlation.

##### ppRNFL, mRNFL and GCL thickness measurements

**Fig. 1:**
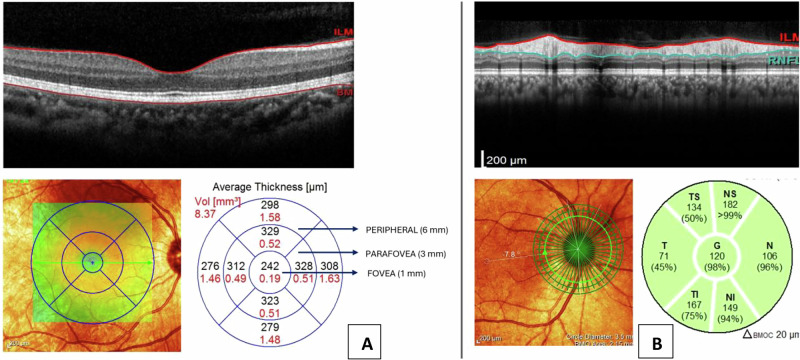
**A** Macular thickness measurements in the nine Early Treatment of Diabetic Retinopathy Study (ETDRS) zones viz. In all, 6 mm peripheral macular (divided into S, T, I and N); 3 mm depicting the parafovea (divided into T, S, N and I) and the central 1 mm representing the fovea. **B** Peripapillary retinal nerve fibre layer (ppRNFL) and the Garway Health sectors, i.e., NS, TS, T, TI, NI, N and G. (Source: author’s own creation) S superior, I inferior, N nasal, T temporal, NS superior nasal, TS superior temporal, TI inferior temporal, NI inferior nasal, G global.

The foveal, parafoveal and peripheral mRNFL and GCL thicknesses were measured using the fast retina scan on the Heidelberg Spectralis OCT (Heidelberg Engineering, Germany; https://business-lounge.heidelbergengineering.com). The macula is imaged in a high-resolution volumetric pattern in a 20 × 20° raster scan area consisting of 25 horizontal line scans with 1024 A-scans per line. The thickness was displayed in the nine areas of the Early Treatment Diabetic Retinopathy Study (ETDRS) map (Fig. 1A).

Two ppRNFL thickness measurements were obtained. First, the standard ppRNFL scan took 768 A-scans within a 3.5-mm circle diameter centred around the optic nerve head (ONH). Thereafter, the Bruch’s Membrane Opening ppRNFL (BMO-ppRNFL) scan was obtained using the Glaucoma Module Premium Edition application. An A-scan pattern from 24 radially equidistant B-scans, each with 1536 A-scans and subtending 15°, centred on the ONH with diameters of 3.5, 4.1 and 4.7 mm were taken. Here, the thickness of the neuroretinal rim was analysed based on the minimum rim width, which is the distance from the inner limiting membrane (ILM) to the BMO. Only the 3.5 mm diameter was used for analysis. BMO is a more anatomically accurate reference point for measuring RNFL thickness because it corresponds with the termination of the optic nerve fibres at the ONH, whereas the standard ppRNFL measurement uses the optic disc margin as a reference point, which may not align precisely with the true endpoint of the nerve fibres [18]. For both ppRNFL scans, normative values based on the Spectralis OCT database were automatically provided and represented on the seven Garway health sectors (Fig. 1B). For all OCT measurements, three scans were acquired and only the scans with the highest quality index (≥ 30) were used for analysis.

### Ethical Considerations

Ethical clearance was obtained from the Biomedical Research and Ethical Committee (BREC) at the University of KwaZulu-Natal (reference: BREC/00005507/2023). Informed consent from the participants was obtained. The researchers ensured that all the participants’ information was kept confidential and that the study was conducted in accordance with the principles of the Declaration of Helsinki.

### Data Analysis

Data were entered into Microsoft Excel (https://www.microsoft.com) and was further analysed using SPSS version 28 (https://www.ibm.com) and STATA software version 17 (https://www.stata.com). The data were checked for normality using the Shapiro–Wilk test. To compare RNFL and GCL thicknesses between both groups, the independent *t* test/Mann–Whitney *U* test was employed. Multivariate linear regression analysis was used to determine the effects of AL, SE and age on RNFL and GCL thicknesses.

## Results

There were 122 participants with completed datasets for this study, of whom the eye with the least myopic SER was used for analysis. Participants were divided equally into low myopes (*n* = 61) and near-emmetropes (*n* = 61). The mean age for the entire sample was 20.50 ± 1.86 years (18–27 years), of whom 66 (54.10%) were males, 56 (45.90%) were females, 96 (78.7%) were of African ethnicity and 26 (21.3%) were of Indian ethnicity. The mean SER for near-emmetropes and low myopes was −0.25 D ± 0.25 D (0 to −0.50) and −1.25 D ± 0.57 D (−0.75 to −3.00), respectively. The mean AL for emmetropes and low myopes was 23.41 mm ± 0.77 mm and 23.76 mm ± 0.82 mm, respectively (Table 1). The scan quality index for all OCT scans was between 30 and 48 in both groups.

**Table 1:** Summary of sample characteristics.

	Emmetropes, *n* = 61, mean ± SD	Low myopes, *n *= 61, mean ± SD
Age (years), *n* = 122	20.50 ± 1.86	20.50 ± 1.86
Spherical equivalent (D)	−0.25 ± 0.25	−1.25 ± 0.57
AL (mm)	23.41 ± 0.77	23.76 ± 0.82

*AL* Axial length.

### Comparisons of Retinal Thickness between Emmetropes and Low Myopes

Table 2 shows a comparison of mRNFL between the near-emmetropes and low myopes. The inner temporal aspect of the RNFL at the macula showed a statistically significant reduction in thickness in low myopes (*p* = 0.02). All other comparisons were not significant.

**Table 2:** Comparison of macula retinal nerve fibre layer (mRNFL) thickness (microns) between low myopes and near-emmetropes using the independent *t* test and Mann–Whitney *U* test (*).

mRNFL		Near-emmetropes, *n* = 61, mean ± SD	Low myopes, *n* = 61, mean ± SD	Corrected *p* value (Holm–Bonferroni)
Fovea (central)		10.92 ± 2.43	10.66 ± 2.80	0.58
Parafovea (inner 3 mm)	Superior	23.20 ± 3.46	22.66 ± 2.46	>0.99
	Nasal	19.75 ± 1.99	19.54 ± 1.86	>0.99
	Inferior	23.92 ± 2.98	23.85 ± 3.15	>0.99
	Temporal	16.57 ± 1.15	16.07 ± 1.25	0.08
Peripheral macular (outer 6 mm)	Superior	37.33 ± 4.69	36.11 ± 3.67	0.44
	Nasal	45.77 ± 5.90	44.59 ± 4.90	0.48
	Inferior	38.82 ± 6.20	38.36 ± 4.90	0.65*
	Temporal	17.87 ± 1.23	17.59 ± 0.94	0.48

Table 3 shows a comparison of macular ganglion cell layer (mGCL) between low myopes and near-emmetropes. The inferior (*p* = 0.03) and temporal (*p* = 0.01) aspects of the outer mGCL showed a statistically significant reduction in thickness in low myopes. All other comparisons were not significant.

**Table 3:** Comparison of macular ganglion cell layer (mGCL) thickness (microns) between low myopes and near-emmetropes using an independent *t* test and Mann–Whitney *U* test (*).

mGCL		Near-emmetropic, *n* = 61, mean ± SD	Low myopes, *n* = 61, mean ± SD	*p* value
Fovea (central)		12.90 ± 3.47	12.26 ± 3.75	0.33
Parafovea (inner 3 mm)	Superior	54.48 ± 4.85	54.10 ± 4.38	0.65
	Nasal	52.10 ± 5.99	51.07 ± 5.45	0.32*
	Inferior	53.13 ± 5.53	53.25 ± 4.86	0.90
	Temporal	48.11 ± 5.94	48.85 ± 5.09	0.46
Peripheral macular (outer 6 mm)	Superior	38.15 ± 3.24	37.28 ± 5.43	0.09*^#^
	Nasal	42.48 ± 3.40	41.70 ± 4.45	0.11
	Inferior	36.87 ± 4.35	35.90 ± 3.91	0.03*^#^
	Temporal	41.44 ± 4.46	38.69 ± 4.55	**0.01**^**#**^

^#^Holm–Bonferroni correction applied, bold values indicate *P* ≤ 0.05.

Table 4 shows comparison of the ppRNFL and the BMO-RNFL between low myopes and near-emmetropes. All comparisons were not significant after Holm–Bonferroni corrections were applied.

**Table 4:** Comparison of peripapillary retinal nerve fibre layer (ppRNFL) thickness (microns) and Bruch’s membrane opening (BMO) retinal nerve fibre layer (RNFL) thickness (microns) between low myopes and near-emmetropes using the independent *t* test and Mann–Whitney test (*).

	Near-emmetropes, *n* = 61, mean ± SD	Low myopes, *n* = 61, mean ± SD	*p* value
ppRNFL			
Global (G)	108.74 ± 10.51	106.18 ± 11.68	0.21
Superior nasal (NS)	134.44 ± 24.58	123.66 ± 23.24	0.06^#^
Nasal (N)	76.49 ± 14.06	79.46 ± 20.28	0.35*
Inferior nasal (IN)	131.59 ± 26.19	129.52 ± 32.17	0.70
Inferior temporal (IT)	150.33 ± 20.26	144.56 ± 20.74	0.12
Temporal (T)	75.18 ± 12.66	71.84 ± 11.00	0.12*
Superior temporal (ST)	151.70 ± 24.38	148.90 ± 19.00	0.48
BMO-RNFL			
Global (G)	110.31 ± 11.97	108.03 ± 13.40	0.32
Superior nasal (SN)	148.92 ± 28.31	135.26 ± 31.17	0.06^#^
Nasal (N)	91.92 ± 16.44	91.16 ± 18.45	0.81*
Inferior nasal (IN)	132.95 ± 26.41	133.51 ± 31.08	0.92
Inferior temporal (IT)	159.13 ± 22.20	156.31 ± 20.80	0.47
Temporal (T)	74.13 ± 9.64	71.74 ± 15.43	0.31*
Superior temporal (TS)	137.64 ± 22.74	138.39 ± 20.25	0.85

^#^Holm–Bonferroni corrected.

**Fig. 2:**
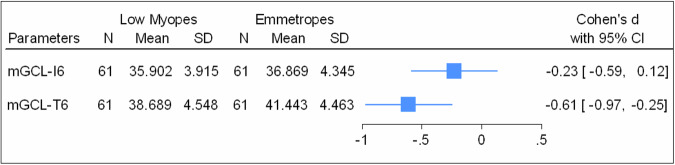
Forest plot showing Cohen’s *d* effect size comparisons for the zones that were significantly thinner in low myopes. Mean difference: mGCL-S6: −0.87 (95% CI: −2.47 to 0.74), mGCL-I6: −0.97 (95% CI: −2.45 to 0.52), mGCLT6: −2.75 (95% CI: -4.37 to −1.14). mGCL macular ganglion cell layer, I6 inferior 6 mm, T6 temporal 6 mm.

Figure 2 shows the effect size comparisons for zones of the mGCL that showed statistically significant thinning in low myopes using the Cohen’s *d* test, which compared the thickness mean values between these zones. The greatest thinning occurred at the temporal aspect of the outer mGCL, with an effect size of −0.61, suggesting a moderate effect size and clinical relevance.

### Association of RNFL and mGCL in Low Myopes

The associations between AL, SER and age with retinal thickness variables of mGCL, mRNFL, ppRNFL and BMO-RNFL for the low myopes were obtained using a multivariate linear regression.

#### Association between AL and Retinal thickness

Tables 5 and 6 show the association between AL and mGCL, mRNFL, ppRNFL and BMO-RNFL thickness in low myopes and near-emmetropes when the age, SER and gender were controlled.

**Table 5:** The association between AL and mRNFL and mGCL thickness in low myopes and near-emmetropes.

Retinal location		Beta-coefficient	95% CI	*p* values	Beta-coefficient	95% CI	*p* values	Effect size *p* value* (exp. vs control)
		Low myopes			Emmetropes			
mRNFL								
Fovea (central)		0.20	−0.17 to 0.58	0.28	0.34	0.04–0.65	**0.03**	**0.01**
Parafovea (inner 3 mm)	Superior	0.79	0.45–1.14	**<0.001**	1.23	0.81–1.64	**<0.001**	**–**
	Nasal	0.93	0.68–1.18	**<0.001**	0.70	0.46–0.94	**<0.001**	**–**
	Inferior	0.72	0.26–1.17	**0.003**	0.99	0.62–1.35	**<0.001**	**–**
	Temporal	0.70	0.52–0.89	**<0.001**	0.72	0.57–0.87	**<0.001**	**0.01**^**#**^
Macula (outer 6 mm)	Superior	0.84	0.31–1.37	**0.002**	1.75	1.18–2.31	**<0.001**	**0.01**^**#**^
	Nasal	1.15	0.44–1.86	**0.002**	2.08	1.37–2.78	**<0.001**	**–**
	Inferior	0.71	−0.01 to 1.43	0.05	2.16	1.42–2.91	**<0.001**	**0.01**^**#**^
	Temporal	0.46	0.31–0.62	**<0.001**	0.74	0.59–0.89	**<0.001**	**0.01**^**#**^
mGCL								
Fovea (central)		0.76	0.27–1.25	**0.003**	0.55	0.11–0.98	**0.02**	**–**
Parafovea (inner 3 mm)	Superior	1.85	1.19–2.51	**<0.001**	2.31	1.74–2.89	**<0.001**	**0.01**^**#**^
	Nasal	1.75	0.99–2.51	**<0.001**	2.93	1.56–3.03	**<0.001**	**–**
	Inferior	1.64	0.93–2.36	**<0.001**	1.97	1.29–2.65	**<0.001**	**0.04**^**#**^
	Temporal	1.96	1.25–2.68	**<0.001**	2.34	1.65–3.03	**<0.001**	**–**
Macula (outer 6 mm)	Superior	0.39	−0.42 to 1.19	0.34	1.30	0.87–1.73	**<0.001**	**0.01**^**#**^
	Nasal	1.14	0.41–1.88	**0.003**	1.90	1.43–2.37	**<0.001**	**0.01**^**#**^
	Inferior	0.86	0.21–1.50	**0.01**	1.72	1.17–2.27	**<0.001**	**0.01**^**#**^
	Temporal	0.91	0.21–1.61	**0.01**	1.726	1.15–2.30	**<0.001**	**0.01**^**#**^

*AL*axial length, *mRNFL* macular retinal nerve fibre layer, *mGCL* macular ganglion cell layer.

For effect size, comparison between the beta-coefficient of both groups, an independent *t* test was used (*). ^#^Holm–Bonferroni corrected, bold values indicate *P* ≤ 0.05.

**Table 6:** The association between AL and ppRNFL and BMO-RNFL thickness in low myopes and near-emmetropes.

Retinal location	Beta-coefficients	95% CI	*p* values	Beta-coefficients	95% CI	*p* values	Effect size *p* value (exp vs control)
	Low myopes			Near-emmetropes			
ppRNFL							
Global	2.45	0.61–4.29	**0.01**	4.71	3.35–6.07	**<0.001**	**0.02**^**#**^
Superior nasal	6.24	2.74–9.75	**0.01**	6.28	3.17–9.40	**<0.001**	0.06^#^
Nasal	1.52	−1.40 to 4.43	0.30	2.69	0.98–4.39	**0.01**	0.85
Inferior nasal	1.39	−3.14 to 5.92	0.54	6.50	3.17–9.84	**<0.001**	0.25
Inferior temporal	2.62	−0.56 to 5.80	0.11	7.10	4.52–9.68	**<0.001**	0.20^#^
Temporal	1.57	−0.10 to 3.24	0.07	3.56	1.99–5.14	**<0.001**	0.63
Superior temporal	3.11	0.27–5.95	**0.03**	7.09	4.04–10.13	**<0.001**	0.28
BMO-RNFL							
Global	2.64	0.49–4.79	**0.02**	0.37	3.42–6.41	**<0.001**	0.24^#^
Superior nasal	4.35	−0.44 to 9.14	0.07	7.84	4.21–11.46	**<0.001**	0.69
Nasal	3.44	0.63–6.25	**0.02**	4.13	2.13–6.13	**<0.001**	0.71^#^
Inferior nasal	2.84	−1.84 to 7.52	0.23	5.00	1.62–8.33	**0.004**	0.06
Inferior temporal	2.71	−0.53 to 5.95	0.10	7.60	4.89–10.30	**<0.001**	0.13
Temporal	−0.18	−2.31 to 1.95	0.87	3.44	2.25–4.63	**<0.001**	0.24
Superior temporal	0.68	−2.10 to 3.46	0.63	7.27	4.52–10.02	**<0.001**	0.37

*AL*axial length, *ppRNFL* peripapillary retinal nerve fibre layer, *BMO-RNFL* Bruch’s membrane opening-retinal nerve fibre layer.

^#^Holm–Bonferroni corrected; bold values indicate *P* ≤ 0.05.

Table 5 shows significant positive associations for mRNFL in both groups that occurred in the parafoveal (ETDRS 3 mm) aspects of the superior, nasal, inferior and temporal zones with AL. Significant positive associations for the mRNFL ETDRS 6 mm diameter (outer macula) with AL for both refractive groups occurred at the superior, nasal and temporal zones.

The mGCL thickness had a significant positive association at the fovea (ETDRS 1 mm diameter), the inner and the outer mGCL in both near-emmetropes and low myopes, as shown in Table 5. Significant positive associations between mGCL thickness and AL occurred in all sectors of the parafovea, i.e., superior, nasal, inferior and temporal. Significant positive associations for the outer mGCL with AL occurred in the nasal, inferior and temporal sectors.

A notable observation was the strength of these associations, which was weaker in the low myopes. This suggests that AL has a reduced positive association with thickness in these mRNFL and mGCL zones, when compared with the near-emmetropes. To study this effect of these associations, the independent *t* test was used to compare the significant beta-coefficient between both groups, shown in Table 5 (last column). Significant differences for mRNFL occurred in the foveal centre and temporal aspects of the inner mRNFL (ETDRS 3 mm diameter) as well as the superior, inferior and temporal zones of the outer mRNFL (ETDRS 6 mm diameter). Significant differences for mGCL occurred in the superior and inferior zones of the inner mGCL as well as the superior, nasal, inferior and temporal zones of the outer mGCL.

Table 6 shows the association between AL and PP and BMO-RNFL thickness in low myopes and near-emmetropes. Significant positive associations occurred with the global ppRNFL thickness for both groups. No significant associations occurred between AL and BMO-RNFL thickness in both low myopes and near-emmetropes. Similar to mRNFL and mGCL, the strength of these associations was also weaker in low myopes than near-emmetropes, as shown when the significant beta-coefficients were compared using the independent *t* test in Table 6 (last column) which illustrates the effect size.

#### SER and retinal thickness associations

The multivariate linear regression between SE and mRNFL, mGCL, ppRNFL and BMO-RNFL thickness, independent of age, gender and AL showed no significant associations for the low myopia group in all zones.

## Discussion

This study demonstrated that young adults with low myopia exhibited zonal thinning of the mGCL, particularly in the inferior and temporal zones. Significant associations, although weaker, were observed between AL and retinal layer thicknesses, mostly in the macular GCL, RNFL and the global ppRNFL zones. These findings suggest that even low levels of myopia may be associated with early structural compromise of retinal integrity.

Prior studies [7,19] have reported thinning of retinal layers with increasing myopia, predominantly linked with axial elongation and higher RE. However, these investigations contrasted mainly low with moderate and high myopia, leaving a paucity of evidence regarding retinal changes specific to low myopes. The present findings attempted to address this gap by focusing on young adults with low myopia compared with near emmetropia, demonstrating measurable thickness changes at low magnitudes of myopia.

The present study is in agreement with Mwanza et al. [20], who reported thinning of the mGCL in myopia. In the current sample, statistically significant reductions were most evident in the inferior sectors of the outer mGCL, with the temporal aspect showing the most pronounced thinning. This is consistent with Seo et al. [21], who found the greatest thinning in the temporal mGCL, associated with increasing SER in myopia. In contrast, much of the existing literature has concentrated on RNFL changes. For instance, Leung et al. [22] and Kang et al. [23] reported reductions in mRNFL thickness, especially in the superior and inferior quadrants; findings that diverge from the present results, where thinning was most prominent in the temporal sector. Such differences may possibly reflect variations in ethnicity (Asians vs Africans), the degree of myopia (low vs high) and age ranges, as some prior studies included participants up to 60 years of age.

With respect to ppRNFL, no significant changes were observed here in any zone. However, this disagreement with Zha et al. [24], who reported thinning in the superior, inferior and nasal quadrants of myopic eyes. Malakar et al. [25] found thinning in both nasal and temporal quadrants, with greater reductions temporally, in a Korean cohort. These discrepancies may also highlight the influence of ethnicity, refractive range and methodology.

The structural thinning observed in low myopes may be explained by the early vulnerability of RGCs. Their dendrites, located in the inner plexiform layer, integrate inputs from bipolar and amacrine cells and transmit signals via axons through the optic nerve [26]. Integrity of these dendrites is essential for visual processing, and degeneration, as in myopic maculopathy, leads to irreversible vision loss. The current finding of thinning in low myopes indicates that myopia represents potential retinal disruptions even at low magnitudes, raising the concern that preclinical changes may precede functional deficits.

The temporal mGCL may be particularly susceptible because it is the last region to mature during retinal development [27]. Delayed maturation may render it structurally more fragile, consistent with the observation of temporal thinning. Further, Jin et al. [28] reported thinner retinal layers in myopic children and adolescents, suggesting that retinal thinning may arise from interference with retinal development rather than progressive loss in adolescence. Early-onset myopia, especially before age 10, may predispose individuals to thinner retinas in adulthood. The present study of young adults did not assess myopia onset, but this remains an important consideration for future research.

Axial elongation is another plausible mechanism. A weaker but significant positive association between AL and mGCL and mRNFL was observed here, which is in agreement with Hirasawa et al. [29] and Chua et al. [30]. Higashide et al. [31] suggested that scleral stretching from elongation particularly affects the outer macular regions, which may explain the regional patterns observed. Notably, the current study also found positive associations between AL and global ppRNFL thickness, in partial agreement with Yoo et al. [32], although not in agreement with Rauscher et al. [33]. The weaker correlations in low myopia suggest that axial elongation exerts subtler influences at lower levels of RE. This may also provide a working hypothesis in future studies of young adults with low myopia.

In contrast, the SER showed no significant associations with mRNFL or mGCL in the low myopic sample. This differs from Budenz et al. [34] and Rauscher et al. [33], who reported RNFL loss with increasing myopia. However, the present sample had a strict focus on low myopia, which may account for this discrepancy. Furthermore, no associations between SER and ppRNFL, BMO-RNFL, mGCl or mRNFL were observed. While Salih et al. [35], showed linear correlations in the superior and inferior ppRNFL with SER in low myopes, such disagreements indicate that more research is required to reach consensus.

The mGCL plays a central role in visual function, and thinning of this layer has been shown to precede measurable visual field loss [36,37]. Localised thinning could therefore impair central visual tasks such as contrast sensitivity or colour discrimination. The current results suggest that low myopia may not be entirely benign and should be considered in early monitoring even in young adults. However, the absolute magnitude of thinning in this study was modest, with small to moderate effect sizes. This does not, by itself, establish current functional deficits or predict future pathology. Therefore, clinical implications should be approached cautiously, and functional assessments such as microperimetry and contrast sensitivity testing, combined with longitudinal follow-up, may be needed to determine whether structural changes translate to functional impairment.

Ethnic variability must also be considered. Tham et al. [38] reported a thinner ganglion cell-inner plexiform layer (GCIPL) in Indians compared with Chinese and Malays. Banghart et al. [39] found thinner mRNFL and GCIPL in Asian and Black participants relative to Caucasians, differences that disappeared after accounting for total retinal thickness. However, Davey et al. [40] found no significant ethnic differences. Given that most studies on myopic thinning have been conducted in East Asians, the current findings in a predominantly African cohort highlight the importance of ethnicity-specific normative databases for accurate interpretation and management.

### Limitations

This study has several limitations. A major limitation of the study was that non-cycloplegic autorefraction was utilised, potentially allowing pseudomyopia. The accommodative response was measured to attempt to mitigate this risk. Transverse magnification was not adjusted for AL, which may influence thickness measurements, although corneal curvature was accounted for. The isolated GCL zones as shown in the Forest plots in Fig. 2 cannot be accounted for by the Spectralis in its reproducibility estimates for this specific layer, but does estimate for a central foveal thickness (b-scan) of 2.4 microns (Heidelberg Engineering, 2022) [41]. This contains the GCL, which may fall below this magnitude. This suggests that the observations may lie outside the instrument noise. The sample had a narrow age range, thus limiting generalisability across the lifespan. The purposive sampling strategy, while appropriate for targeting low myopes, also restricted generalisability and recruitment from clinical settings may introduce selection bias. Finally, reliance on strict inclusion criteria may reduce diversity within the sample.

### Recommendations

Despite these limitations, these findings underscore the importance of recognising structural retinal changes, even in low myopia. Clinically, we recommend incorporating axial biometry and OCT imaging into care for low myopes and young adults to detect structural compromise. Future studies should investigate larger, multi-ethnic cohorts across a broader age range, especially paediatric populations, to better understand the developmental contributions to retinal thinning. Moreover, longitudinal studies are needed to establish whether structural thinning in low myopia progresses and predicts functional deficits. The current study, one of the few in an African cohort, highlights the need to broaden the global myopia conversation beyond Asian and Caucasian populations, and to ensure that low myopia is included in myopia management strategies.

## Conclusion

Thinning of the macular GCL in young adults with low myopia indicates structural alterations and sub-clinical changes associated with low-magnitude myopia. This suggests that myopia represents a visual morbidity, even at a preclinical stage. The study builds on evidence that myopia is not solely a refractive issue and highlights the concern that retinal layer alterations are evident even in young adults with low myopia. Therefore, myopia control should not be restricted to higher degrees of myopia, but rather extended to populations beyond the paediatric age group. Future longitudinal studies are needed to determine whether these differences predict progression or confer an increased risk of visual morbidity.

## Data Availability

All generated data are available upon request.
